# CT Scans and Cancer Risks: A Systematic Review and Dose-response Meta-analysis

**DOI:** 10.1186/s12885-022-10310-2

**Published:** 2022-11-30

**Authors:** Chun-Feng Cao, Kun-Long Ma, Hua Shan, Tang-Fen Liu, Si-Qiao Zhao, Yi Wan, Jun-Zhang, Hai-Qiang Wang

**Affiliations:** 1 Department of Orthopedics, Yongchuan Hospital of Chongqing Medical University, Hua Road, No. 439, Yongchuan, 402160 Chongqing, People’s Republic of China; 2 Institute of Integrative Medicine, Shaanxi University of Chinese Medicine, Xixian Avenue, Xixian District, Xi’an, 712046 Shaanxi Province People’s Republic of China; 3 Department of Orthopedics, No.1 Hospital of Xi’an City, Northwestern University, Xi’an, 710002 Shaanxi Province People’s Republic of China; 4 Department of Health Services, Fourth Military Medical University, Xi’an, 710032 No.169 West Changle Road, Shaanxi Province People’s Republic of China; 5 Baoji Central Hospital, 8 Jiangtan Road, Baoji, 721008 Shaanxi Province People’s Republic of China; 6 School of Public Health, Xi’an Jiaotong University Health Science Center, Xi’an, 710061 Shaanxi Province People’s Republic of China

**Keywords:** Computed tomography, Adult, Cancer risk, Radiation exposure, Meta-analysis

## Abstract

**Background:**

There is still uncertainty on whether ionizing radiation from CT scans can increase the risks of cancer. This study aimed to identify the association of cumulative ionizing radiation from CT scans with pertaining cancer risks in adults.

**Methods:**

Five databases were searched from their inception to November 15, 2020. Observational studies reporting cancer risks from CT scans in adults were included. The main outcome included quantified cancer risks as cancer case numbers in exposed/unexposed adult participants with unified converted measures to odds ratio (OR) for relative risk, hazard ratio. Global background radiation (2.4 mSv per year) was used as control for lifetime attribution risk (LAR), with the same period from incubation after exposure until survival to 100 years.

**Results:**

25 studies were included with a sum of 111,649,943 participants (mean age: 45.37 years, 83.4% women), comprising 2,049,943 actual participants from 6 studies with an average follow-up period as 30.1 years (range, 5 to 80 years); 109,600,000 participants from 19 studies using LAR. The cancer risks for adults following CT scans were inordinately increased (LAR adults, OR, 10.00 [95% CI, 5.87 to 17.05]; actual adults, OR, 1.17 [95%CI, 0.89 to 1.55]; combined, OR, 5.89 [95%CI, 3.46 to 10.35]). Moreover, cancer risks elevated with increase of radiation dose (OR, 33.31 [95% CI, 21.33 to 52.02]), and multiple CT scan sites (OR, 14.08 [95% CI, 6.60 to 30.05]). The risk of solid malignancy was higher than leukemia. Notably, there were no significant differences for age, gender, country, continent, study quality and studying time phrases.

**Conclusions:**

Based on 111.6 million adult participants from 3 continents (Asia, Europe and America), this meta-analysis identifies an inordinately increase in cancer risks from CT scans for adults. Moreover, the cancer risks were positively correlated with radiation dose and CT sites. The meta-analysis highlights the awareness of potential cancer risks of CT scans as well as more reasonable methodology to quantify cancer risks in terms of life expectancy as 100 years for LAR.

**Prospero trial registration number:**

CRD42019133487.

**Supplementary Information:**

The online version contains supplementary material available at 10.1186/s12885-022-10310-2.

## Background

As an auxiliary diagnostic imaging modality, CT scans play an important role in the diagnosis of various complicated diseases [1]. As new clinical indications continue to be discovered, the use of CT scan has increased rapidly over the past decade worldwidely [2–5]. Although CT scans are of great diagnostic benefits to individual patients, relatively higher radiation doses are delivered compared with other conventional imaging modalities [1, 6, 7]..

Substantial follow-up works are now underway to determine whether patients experiencing ionizing radiation from CT scans can significantly increase the risk of cancer. Indeed, many lines of evidence have shown that ionizing radiation from CT scans is associated with cancer/tumor risks [8–12]. Whereas recently epidemiological studies have examined potential disease risks from pediatric CT scanning, studies of cancer risk from CT scans during adulthood are also of critical important for public health. Adults receive over 10 times more CT scans than children [13–15] and most radiation induced cancers occur during middle or older age.

However, researchers questioned the validity of the earlier indirect estimates based on uncertain risk projections for radiation [16], whereas others declared that the findings of recent CT scan studies need to be interpreted with caution, due to the possibility of reverse causation [17]. Therefore, we cannot necessarily attribute all excess cases of cancer to ionizing radiation during the period of follow-up after patients undergoing CT scans, since the decision of performing CT scans is not allocated randomly but based on medical indications. For example, patients with precancerous symptoms or early symptoms that might prompt their physician to perform a CT scan, which may lead to the possibility of reverse causation. Until now, there is still uncertainty on whether ionizing radiation from CT scans can increase the risks of cancer. It can be hard for the medical community to design and conduct studies to validate previous reports. Prior studies have presented the evidence on cancer and mortality risks of young scoliosis patients from cumulative radiographic radiation (spinal radiographs) [18] and tentatively proposed low radiation X-ray methodology [19], focusing on the issue dynamically with the medical community [20–24] with the aims for benefiting global patients and the public. Consequently, this work aims to evaluate whether adult CT scan exposure can increase the risks of cancer during the follow-up observation, based on systematic review and meta-analysis of global observational studies.

## Methods

### Protocol

This systematic review and meta-analysis was successfully registered in the International Prospective Register of Systematic reviews (PROSPERO) on 28 April 2019, with the Registration number CRD42019133487. The initial enrollment was to study the relationship between CT screening and cancer risk in children and adolescents. We revised the registry for adult CT scans and cancer risks due to a published study by another group during our studying process [25]..

### Study types

Observational studies, cohort studies, and case-control studies were included in this systematic review and meta-analysis, while studies assessed review articles, proceedings, case series and case reports were excluded. No restrictions to the language of publication were applied to select primary studies. We added extra studies by additional hand search of the reference lists of related articles.

### Population

Patients meeting the following requirements were included in this study: 1) Patients were older than 18 years for their first CT scan, 2) Patients did not have any malignant diseases (such as precancerous symptoms or early symptoms of the cancer) prior to their first CT scan, 3) Patients with follow-up of more than 1 year after their first scan. 4) Patients underwent at least one CT scan. Those with the following conditions were excluded: 1) Patients with incomplete demographic data or data errors, 2) Patients lost follow-up for a variety of factors.

### Interventions

Included patients received one or more electronically archived CT scans. The absorbed dose from a CT scan mainly depends on factors including age, sex, examination site, year of scan (2007 as a milestone for CT scans in terms of dose reduction in Annals of the ICRP [26]), and machine parameters.

### Outcome measures

The study included two outcome measures.

1. Cancer risks, including each part of the human body with at least 1 year lag period after first exposure.
2. The potential associations between adult CT scan exposure radiation doses and cancer risks, including lifetime attribute risk (LAR).

### Search methods for identification of studies

A thorough search was conducted in the following global databases, PubMed, Medline, EMBASE, Web of Science, Springer Link, Cochrane Library, from inception through Nov 15th 2020 with terms including “Adult”, “computerized tomography, X ray”, and “Cancer”. Search strategies were designed by an experienced librarian and revised by another librarian according to the PRESS (Peer Review of Electronic Search Strategies) checklist [27]. Three reviewers performed the selection process independently, and disagreements were discussed and resolved by a fourth reviewer.

### Assessment of risk of bias (ROBs) in included studies

The ROBs of each study were assessed independently by two authors according to Newcastle-Ottawa Scale [28]..

### Dealing with missing data

When missing data were encountered in included articles, we contacted the corresponding authors of relevant studies for these data by sending electronic mails twice with time interval of 1 month. When the study did not provide the number of cancer cases in the case/exposed group or control/unexposed group, the numbers were calculated by the formula defined as R_e_/R_u_ for RR (Relative Risk), R_e_ (1- R_u_)/[R_u_ (1- R_e_)] for OR (the rate in exposed persons, denoted R_e_; the rate in unexposed persons, denoted R_u_), and (O_r_/O_e_)/(C_r_/C_e_) for HR (experimental group, denoted O; control group, denoted C; actual persons, denoted r; theoretical persons, denoted e) [29].

### Assessment of publication bias

Publication bias were explored by a funnel plot (i.e., plots of study results against precision), Egger’s tests [30]..

### Subgroup analysis

In studies using LAR to estimate cancer risks, control group was defined according to the global incidence of cancer at 2.4 mSv of background radiation dose, i.e., lifetime baseline risk (LBR) [31]. We used the radiation risk models for sex- and organ-specific cancer incidence developed by the National Research Council’s BEIR VII committee [32]. LAR calculates the risk of cancer from an incubation period after radiation exposure (5 years for solid cancer and 2 years for leukemia) to survival to 100 years. LAR and LBR were calculated with the same method. For an age with a mantissa of 5: 45 years, divide by 2 the sum of the LAR values for 40 years and 50 years. For example, the LAR at the age of 45 years is (141 + 70)/2 = 105.5 at full 100 mSv (141 cases per 100,000 at 40-year-old and 70 cases per 100,000 at 50-year-old by BEIR VII). If less than 100 mSv, if it was 2.4 mSv, the LAR at the age of 40 years, 45 years, and 50 years are 41(2.4/100), 105.5(2.4/100), and 70(2.4/100), respectively.

### Sensitivity analysis

Sensitivity analysis was performed by removing relevant studies to observe whether the homogeneity and the results change significantly. If the heterogeneity was too large to be analyzed, descriptive analyses were presented.

### Assessment of heterogeneity

All analyses were performed using Stata V.16.0 software (Stata Corp LP, College Station, Texas, USA). Heterogeneity among primary studies were analyzed using standard Chi-squared tests (*P* value) and the *Ι*^*2*^ statistic as recommended by the Cochrane Handbook for Systematic Reviews of Interventions [33]. We interpreted *Ι*^*2*^ values according to Deeks [34]. Meta-regression was used to explore prior factors that may be important sources of heterogeneity. *P* < 0.05 was considered as a statistically significant.

## Results

### Hallmarks of included studies

**Fig. 1:**
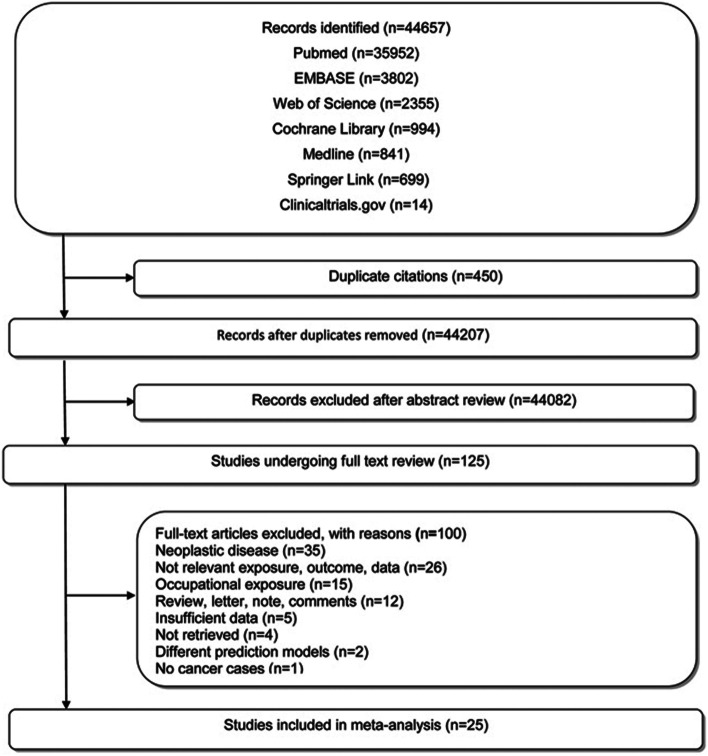
Flowchart of literature screening process

The literature-retrieving strategy and pertaining results were shown in Fig. 1 and Table S1. A total of 44,657 relevant studies were preliminarily reviewed. In total, 125 studies satisfied the eligibility criteria for full-text screening and 25 were eventually included for this meta-analysis [2, 8–11, 35–54]. Of these 25 studies, 5 reported actual cancer cases [8, 10, 11, 35, 36], 19 estimated cancer cases by calculating LAR [2, 9, 37–53], 1 reported expected cancer cases in control group [54], and all were published since 2007. Four studies were excluded due to incomplete data [12, 55–57]. Additional data were requested by contacting the authors of 4 studies via repeated electronic mails with a time interval of 1 month (Table S2). However, no replies and requested data were received.

### Participant hallmarks

Studies were predominantly from the US (*n* = 11), followed by China (*n* = 4), Sweden (*n* = 3), Greece (*n* = 2), and Canada, Italy, Thailand, Croatia, and Denmark (n = 1 each); One study [42] included populations from China (Hong Kong) and the US. Based on the LAR base number of 100,000 persons, 25 studies were included with a sum of 111,649,943 participants, comprising 2,049,943 actual participants (111,449 cases VS 1938494 controls) from 6 studies; 109,600,000 estimated participants (54,912,051 participants received at least one CT radiation) from 19 studies. The average age was 51.3 years in CT scan exposed group (range, 20.0 to 94.0 years) and 40.5 years in control group (range, 25 to 84 years). The gender was reported in 19 studies of CT scan exposed group and 5 studies of unexposed group. The proportion of women was even higher (53.0%, 140,305 participants in CT group; 85.6%, 1,914,381 participants in unexposed group). The average follow-up time was 12.1 years in actual group (range, 5.9 to 22.5 years), 48.0 years (range, 5 to 80 years) in LAR group. The radiation dose per capita was 66.7 mSv (range:5.15 mSv to 122 mSv). A detailed list of study hallmarks was shown in Table 1 and Table 2.

**Table 1:** Detailed information and characteristics of participants

Study	Country	Participants		Age (Y)		Gender (F/M)		Cancer Case		Participants/Cancer case	
		E	U	E	U	E	U	E	U	LAR	LBR
Burton [ 35], et al. 2018	Canada	5859	1,292,059	30.4 ± 5.8	28.7 ± 5.7	F:5859	F:1292059	27	10,080	–	–
Nordenskjöld [ 10], et al. 2017	Sweden	15,534	51,398	41.27	38.88	7786 7748	26,296 25,102	23	45	–	–
Hung [ 36], et al. 2013	China	18,697	19,109	62.5 ± 13.9	60.2 ± 14.8	8330 10,367	10,799 8310	954	885	–	–
Olsen [ 54], et al. 2014	Denmark	15,104	15,104	NA	NA	NA	NA	37	NA	–	–
Shao [ 8], et al. 2020	China	56,050	560,491	> 25	> 25	31,857 24,193	309,561 241,921	2552	17,613	–	–
Davis [ 11], et al. 2011	America	205	333	48.5 ± 12.3	49.8 ± 12.5	91 114	178 155	56	72	–	–
Rampinelli [ 9], et al. 2017	Italy	5203	NA	> 50	NA	1764 3439	NA	–	–	1,600,000 653	1,600,000 136.9
Kritsaneepaiboon [ 37], et al. 2016	Thailand	328	NA	40.10 ± 15.81	NA	73 255	NA	–	–	100,000 140	100,000 18.4
Griffey [ 38], et al. 2009	America	130	NA	56	NA	82 48	NA	–	–	100,000 1219.5	100,000 14.4
Einstein [ 39], et al. 2008	America	50	NA	61 ± 12	NA	20 30	NA	–	–	100,000 62.7	100,000 12.9
Faletra [ 40], et al. 2010	Sweden	749	NA	52–70	NA	280 469	NA	–	–	2,100,000 5306.9	2,100,000 253.1
Niemann [ 41],et al 2013	Sweden	691	NA	66	NA	339 352	NA	–	–	8,700,000 538.6	8,700,000 143.3
Huang [ 42], et al. 2009	China and America	NA	NA	20–80	NA	NA	NA	–	–	23,200,000 19,493	23,200,000 404.1
Perisinakis [ 43], et al. 2015	Greece	136	NA	F:59.4 M:60.6	NA	41 95	NA	–	–	100,000 71.5	100,000 27
Smith-Bindman [ 2], et al. 2009	America	1119	NA	59	NA	535 584	NA	–	–	200,000 423	200,000 51.6
Sodickson [ 44], et al. 2009	America	31,462	NA	56.9 ± 17.5	NA	13,843 17,619	NA	–	–	100,000 300	100,000 28.9
Huang [ 45], et al. 2009	China	NA	NA	20–80	NA	NA	NA	–	–	12,500,000 8690	12,500,000 204.1
Huda [ 46], et al. 2010	America	100	NA	59 ± 11	NA	60 40	NA	–	–	200,000 231	200,000 29.5
Perisinakis [ 47], et al. 2012	Greece	62	NA	F:61 ± 10 M:59 ± 13	NA	31 31	NA	–	–	200,000 59	200,000 25.8
Einstein [ 48], et al. 2007	America	NA	NA	61 ± 12	NA	NA	NA	–	–	800,000 1702.1	800,000 134.9
Kim [ 49], et al. 2009	America	NA	NA	F:45–75 M:55–75	NA	NA	NA	–	–	1,300,000 102	1,300,000 112.7
Majer [ 50], et al. 2018	Croatia	NA	NA	NA	NA		NA	–	–	2,200,000 1410	2,200,000 55.3
Salibi [ 51], et al. 2014	America	67	NA	34 ± 14	NA	54 13	NA	–	–	100,000 810	100,000 39.4
Shah [ 52], et al. 2013	America	24,393	NA	23–84	NA	NA	NA	–	–	200,000 1163.5	200,000 32.7
Wylie [53], et al. 2018	America	3863	NA	20–60	NA	3434 429	NA	–	–	1,000,000 880	1,000,000 199.7

Abbreviations: *E* Exposed, *U* Unexposed, *NA* Not available, *Y* Years, *F* Female, *M* Male, *LAR* Lifetime attributable risks, *LBR* Lifetime background risks

**Table 2:** Detailed information and characteristics of included studies

Study	Study Type	Reason	CT scanner	NO. of CT scan (CT sites, participants)	Effective dose (mSv)	Cumulative dose (mSv)	Observation time	Follow-up (Y)	
								E	U
Burton [ 35], et al. 2018	A	NA	NA	1, 5655 ≥ 2, 204	10–44	NA	1995–2004	5.9	11.1
Nordenskjöld [ 10], et al. 2017	A	NA	NA	NA	NA	NA	1973–1992	22.5	21.9
Hung [ 36], et al. 2013	A	CAD	NA	1–5, 657 ≥ 6, 297	NA	NA	1997–2010	12	12
Olsen [ 54], et al. 2014	A	CHD	NA	NA	NA	NA	1977–2008	8	8
Shao [ 8], et al. 2020	B	NA	NA	1–3, 17,423 ≥ 4, 2742	0.5–17.0	0.5–71.3	2000–2013	9.3- 9.9	9.3- 9.9
Davis [ 11], et al. 2011	B	NA	NA	1–2, 98 ≥ 3, 30	2	1.5–10	2003.04–2007.12	2	2
Rampinelli [ 9], et al. 2017	C	LCS	NA	NA	M: 1.0 F: 1.4	M: 9.3 F: 13	2004–2015	10	10
Kritsaneepaiboon [ 37], et al. 2016	NA	Multiple-injury	64-multislice and 16-multislice CT, Phillips Brilliance	NA	NA	19.43 ± 21.31	2013.01.01- 2013.12.31	1	NA
Griffey [ 38], et al. 2009	C	Emergency	64-multislice CT	NA	NA	122	2005.06.01–2006.05.31	7.7	NA
Einstein [ 39], et al. 2008	C	NA	Siemens AG 16-slice	NA	8.8 ± 2.9	NA	NA	NA	NA
Faletra [ 40], et al. 2010	C	Coronary disease	64-slice CTCA	NA	Low: M 3.9 ± 1.7, F 3.1 ± 1.4 High: M 23.3 ± 4.0, F 22.3 ± 2.3	NA	NA	NA	NA
Niemann [ 41],et al 2013	C	PE	Siemens Sensation 64;Siemens Sensation 16;Siemens Sensation 10	NA	4.35 ± 0.31	NA	NA	1	NA
Huang [ 42], et al. 2009	NA	NA	64-detector CT system (Discovery PET/CT,GE Healthcare, Milwaukee, Wis)	NA	M: 13.65, 24.80, 32.18 F: 13.45, 24.79, 31.91	100	NA	NA	NA
Perisinakis [ 43], et al. 2015	D	NA	256-slice CT	NA	8.9 ± 0.9	NA	NA	NA	NA
Smith-Bindman [ 2], et al. 2009	E	Various	NA	NA	2–31	NA	2018.01.01–2018.05.30	NA	NA
Sodickson [ 44], et al. 2009	A	Cysticfibrosis	NA	NA	NA	54.3	1985–2007	NA	NA
Huang [ 45], et al. 2009	D	NA	NA	NA	45–73	100	NA	NA	NA
Huda [ 46], et al. 2010	C	CAD	64-MDCT scanner (Siemens Healthcare)	NA	25.1 ± 4.9	NA	NA	NA	NA
Perisinakis [ 47], et al. 2012	NA	NA	256-slice TRO-CTA	NA	M: 3.8 ± 0.7 F: 6.5 ± 1.0	NA	NA	NA	NA
Einstein [ 48], et al. 2007	NA	NA	64-slice scanner (SOMATOM Sensation 64, Siemens AG, Munich, Germany)	NA	NA	lungs: 42–91 breast: 50–80	NA	NA	NA
Kim [ 49], et al. 2009	NA	CAC	NA	NA	3.1	NA	2000–2005	NA	NA
Majer [ 50], et al. 2018	NA	NA	NA	NA	NA	16–94	NA	NA	NA
Salibi [ 51], et al. 2014	E	TBI	NA		NA	87 ± 45	NA	NA	NA
Shah [ 52], et al. 2013	C	Emergency	NA		83.4	NA	2001.01–2007.12	NA	NA
Wylie [53], et al. 2018	C	Hip Pain	NA		30	NA	2015.01–2016.12	NA	NA

### ROBs of included studies

The quality scores of 5 studies with actual participants and cancer cases ranged from 7 to 9 (Table S3A). Twenty of the studies were estimated cancer incidence and not case-control or cohort studies, the quality scores ranged from 2 to 6 (Table S3B).

### Cancer risk from CT scans

**Fig. 2:**
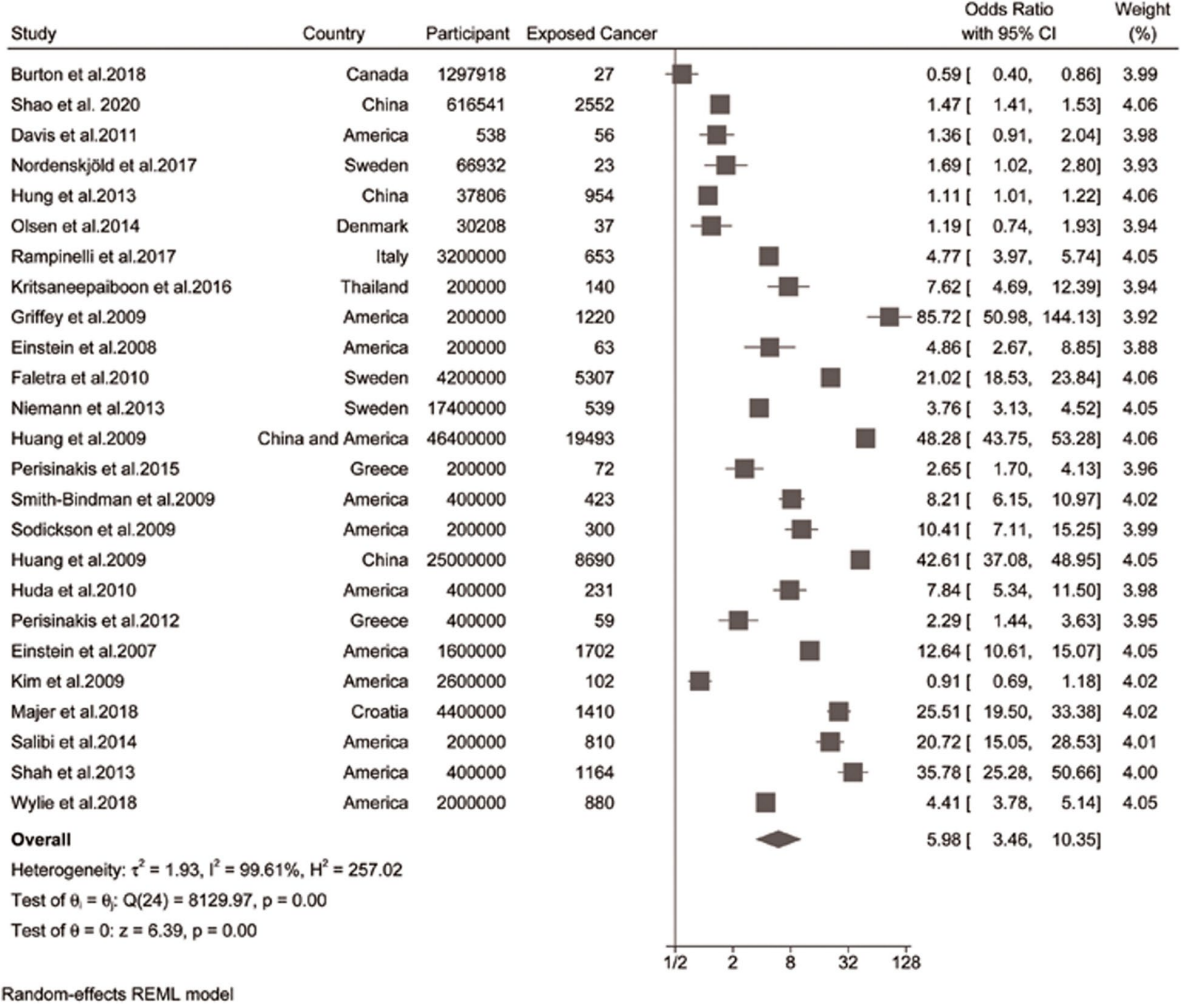
Forest plot of cancer risk from CT scans versus unexposed group

The cancer risks for adults following CT scans were inordinately increased (LAR adults, OR, 10.00 [95% CI, 5.87 to 17.05], *Ι*^*2*^ = 99.14; actual adults, OR, 1.17 [95%CI, 0.89 to 1.55], *Ι*^*2*^ = 94.18; combined, OR, 5.89 [95%CI, 3.46 to 10.35], *Ι*^*2*^ = 99.61%; Fig. 2). Notably, cancer incidence for actual studies was significantly different due to the inclusion (OR, 1.17 [95% CI, 0.89 to 1.55]) or exclusion (OR, 1.32 [95% CI, 1.11 to 1.56]) of Bruton’s study [35] with pregnant women participants (Table 3). Subgroup analysis indicated that cancer risks from LAR studies were significantly higher than actual studies (*P* = 0.00; Table 3), due to a longer life expectancy (to 100 years) used for calculation.

**Table 3:** Subgroup analyses of CT exposure radiation and the risks of cancer incidence

	n	OR (95% CI)	I [2](%), P_heterogeneity_	P_subgroup_
All studies	25	5.98 (3.46 to 10.35)	99.61, 0.00	
Sex				0.71
Men	18	4.89 (2.40 to 9.96)	99.43, 0.00	
Women	18	5.88 (2.96 to 11.68)	99.52, 0.00	
Age				0.93
< 45 years	11	5.79 (2.08 to 16.14)	99.64, 0.00	
45 to 65 years	11	5.04 (2.02 to 12.57)	99.47, 0.00	
> 65 years	8	6.66 (2.07 to 21.38)	98.00, 0.00	
Scanning year				0.88
Before 2007	8	3.83 (1.05 to 13.91)	98.86, 0.00	
After 2007	5	3.39 (1.60 to 7.72)	99.54, 0.00	
Data type				0.00,0.00
Actual	6	1.17 (0.89 to 1.55)	94.18, 0.00	
Actual (Except Burton et al. 2018)	5	1.32 (1.11 to 1.56)	80.79, 0.00	
Estimate	19	10.00 (5.87 to 17.05)	99.14, 0.00	
Country				0.19
NonAmerica	14	4.65 (2.15 to 10.05)	99.73, 0.00	
America	12	9.56 (4.48 to 20.40)	99.03, 0.00	
Continent				0.58
Asia	5	7.81 (1.57 to 38.89)	99.91, 0.00	
Europe	8	4.37 (2.00 to 9.56)	98.74, 0.00	
America	14	7.72 (3.42 to 17.42)	99.19, 0.00	
Cancer type				0.01
Nonleukemia	14	5.57 (2.36 to 13.16)	99.67, 0.00	
Leukemia	5	1.66 (1.29 to 2.15)	19.62, 0.21	
CT scan sites				0.00
< 3	15	3.38 (1.80 to 6.33)	99.23, 0.00	
≥ 3	10	14.08 (6.60 to 30.05)	99.53, 0.00	
Quality scores				0.25
< 5	14	8.04 (4.38 to 14.75)	99.23, 0.00	
≥ 5	11	4.12 (1.57 to 10.86)	99.69, 0.00	

Abbreviations: *OR* Odds ratio, *CI* Confidence interval

There was no evidence of publication bias (egger, *P* = 0.58). Cancer risks of exposed men and women were not significantly different (*P* = 0.71, men, OR, 4.89 [95% CI, 2.40 to 9.96]; *Ι*^*2*^ = 99.43%; women, OR, 5.88 [95% CI, 2.96 to 11.68], *Ι*^*2*^ = 99.52%; Table 3). There were no significantly different cancer risks (*P* = 0.93) for adults in terms of age (Table 3, less than 45 years, OR, 5.79 [95% CI, 2.08 to 16.14], *Ι*^*2*^ = 99.64%; 45 to 65 years, OR, 5.04 [95% CI, 2.02 to 12.57], *Ι*^*2*^ = 99.47%; older than 65 years, OR, 6.66 [95% CI, 2.07 to 21.38], *Ι*^*2*^ = 98.00%).

The LAR estimation included the 2006 National Academy of Sciences BEIR VII. We extracted data in terms of scanning year (before and after 2007, 26] and countries (US or not) to explore the impact of scanning year and countries on cancer risks. Scanning year, countries, and continents of studies did not alter cancer risks (*Ι*^*2*^ = 99.61%, *P* = 0.88*; Ι*^*2*^ = 99.61%, *P* = 0.19; *Ι*^*2*^ = 99.61%, *P* = 0.58, Table 3). There was no significant difference in study quality scores (*Ι*^*2*^ = 99.61%, *P* = 0.25; Table 3).

Dose-response analysis showed that the cancer risks significantly increased linearly with radiation dose of CT scans (Coefficient = 0.03, *P* = 0.00) (Fig. S1). In details, the cancer risks were lowest for radiation dose of less than 15 mSv (OR, 2.51 [95% CI, 1.55 to 4.06], *Ι*^*2*^ = 94.46%), highest for dose greater than 55 mSv (OR, 33.31 [95% CI, 21.33 to 52.02], *Ι*^*2*^ = 97.09%), intermediate for dose of 15 to 55 mSv (OR, 5.48 [95% CI, 2.74 to 10.97]), with significant differences between triple groups (*P* < 0.001, dose grouping according to the overall radiation dose distribution included in the studies) (Fig. S2). Moreover, the relationship between cancer risk and radiation dose was more significant in the < 45 years group (*P* = 0.00), while there was no significant difference in 45–65 years groups (*P* = 0.08, Fig. S3).

For cancer types, a significantly higher risk of non-leukemic cancer was noted (*P* = 0.01; Table 3), in comparison with leukemic cancer. Cancer risks were significantly increased when more than 3 sites were scanned (*P* = 0.00; Table 3).

### Heterogeneity and sensitivity analyses

Sensitivity analyses did not alter the results, by excluding 1 study at a time from each meta-analysis (Table S4). Heterogeneity was present in all analyses (> 99% for all pooled estimates). Meta-regression (Table S5) identified data type, CT sites, and radiation dose contributing to heterogeneity for cancer risk, with no factors substantially reducing heterogeneity between studies.

## Discussion

### Main findings and the significance of using LBR for LAR studies

The meta-analysis presents novel and complete evidence for the common and important issue concerned by clinicians, patients and the public, i.e., exposed CT scans and pertinent cancer risks for adults in their remaining lives. Based on 2,049,943 actual and 109,600,000 LAR/LBR participants from 9 countries, cancer risks for adults undergoing CT scans were successfully identified. Importantly, studies using LAR have been neglected in previous synthesized reports; partly due to the paucity of an appropriate control/unexposed population in quantify OR for these studies.

Indeed, selecting a control group (LBR) for LAR studies was challenging for such meta-analysis with global included studies. Participants unexposed to CT scans receive cosmic radiation (background radiation) in the earth, with various radiation dose range in different regions [31]. When background radiation was greater than 20 mSv per year, the area is designated as high natural background radiation (HNBR) region [58]. An ideal LBR should estimate cancer incidence based on various background radiation doses in different regions. Since HNBR areas were not covered in LAR studies for our meta-analysis, it is appropriate to use the global mean of 2.4 mSv per year as control group to estimate cancer risks. Our data showed that the incidence of cancer in the LBR group (42.7/100000 for men and 65.7/100000 for women) was lower than that in the CT exposed group (68.8/100000 for men and 91.9/100000 for women) and the updated annual global incidence of cancer [59]..

### Sample size strengths and profound analysis for cancer risks from CT scans

So far, no studies have quantified the cancer risk of low-dose radiation. One of the most important reasons is insufficient sample size. According to the evaluation of a total of 1000 mSv for a sample of 1000 people, the sample size needed to evaluate 100 mSv would be 100,000, 10 mSv would be 10 million [60]. In this meta-analysis, the sample size was sufficient with strengths, including 24,000,538 participants for CT radiation exposure < 15 mSv, 1,056,402,315 for 15 to 55 mSv, and 76,600,000 for dose > 55 mSv. Cancer risks increased with cumulative radiation dose from CT scans, consistent with previous evidence [61]. Meanwhile, the results showed that cancer risks increased slowly during radiation dose below 55 mSv, and rapidly for those above 55 mSv.

Ample evidence from Japanese atomic bomb survivors indicates that children are more susceptible than adults to the deleterious effects of ionizing radiation [62]. Children have longer life expectancy since exposure to ionizing radiation, providing more time for a cancer to manifest. This point supports our results with higher cancer risks for LAR than actual studies, since life expectancy used for LAR was 100 years [63]. Notably, there was no significant difference of age on cancer incidence when we divided adults into young, middle-aged and older (age groups according to the 2010 WHO Recommendations on physical activity for health) [64]. Similarly, age did not significantly alter cancer risks for children and adolescents exposed for CT scans in Huang’s study [25] (0 to 5 years (relative risk [RR], 1.35), 6 to 15 years (RR, 1.14), and > 15 years (RR, 1.24) [25]. Mavragani et al. found that DNA damage from low radiation doses was not severe enough to cause cell death, but could trigger a red flag [65, 66]. One of those red flags is the body’s immune system. Increasing evidence suggests that radiation exposure of low dose may not only have immunosuppressive effects, but likely to be associated with a radiation-induced enhancement in the immune system [67–69]. However, immune systems of older adults are not as well functioning as those of younger people. This factor may underlie the results that the risk of cancer from CT radiation does not continue to decrease with age. This may also explain why cancer risks in the 45–65 years group are less sensitive to radiation exposure than those in the < 45 years group. Despite the age differences were insignificant, there was a significant increase in cancer risks for exposed adults compared with the background radiation group.

In 2007, the International Commission on Radiological Protection (ICPR) published a guide to the basic principles of appropriate radiation Protection based on radiation exposure. Since then, almost all international standards and national regulations dealing with radiation protection have been based on the recommendations of the Committee. Consequently, 2007 should be a milestone for CT scan in terms of dose reduction. Even in this study, no significant difference was found between radiation and cancer risk compared with studies published before or after 2007. What is clear is that researchers will adjust their protective strategies to a higher level in the plethora of exposure situations, as ICRP itself states.

### Limitations

Firstly, There are various confounders for cancer incidence [70], including genetics [71], hormone levels, [72, 73] tobacco, alcohol, overweight, physical activity [74, 75], socio-economic status, [76] and infectious agents [77, 78]. These confounders were not included in this meta-analysis, due to the unavailability of information from original studies. Secondly, the numbers of participants in this study are mostly derived from estimated group and tend to be theoretical cancer risks from CT scan. Nevertheless, evidence from these LAR studies should not be ignored for meta-analysis (have been ignored in previous systematic review). Thirdly, over 80% participants in this study were women, with the risk of cancer in women undergoing CT scans during pregnancy remains unclear. Fourthly, the actual and estimated participants were combined. The difference in age at the end of follow-up between the two groups may amplify the actual cancer incidence. Nevertheless, current follow-up time period from actual studies was relatively limited, with incomplete reflected cancer risks from CT scans. Fifth, it is apparent that CT scans were correlated with increased risk of solid cancers, i.e., cancer risk increased with radiation dose and sites scanned. However, the relative increased quantification of scans may be biased due to different weight factors of various organs/tissues of the body. Finally, heart disease was the most common reason for CT scans in included studies, with variation in terms of the severity of patients, the treatment plan, and the duration of follow-up, thus contributing to the inconsistency of included studies.

## Conclusion

Based on 111.6 million adult participants from 3 continents (Asia, Europe and America), this meta-analysis identifies an inordinately increase in cancer risks from CT scans for adults (Additional Video). Moreover, the cancer risks were positively correlated with radiation dose and CT sites. The meta-analysis highlights the awareness of potential cancer risks of CT scans as well as more reasonable methodology to quantify cancer risks in terms of life expectancy as 100 years for LAR.

## Supplementary Information

**Additional file 1 Table S1.** Search strategy and results

**Additional file 2 Table S2.** Contacting authors for additional information

**Additional file 3 Table S3A.** Newcastle-Ottawa Quality Assessment Scale – Case–control studies. (.docx). **Table S3B.** Newcastle-Ottawa Quality Assessment Scale – Cohort studies

**Additional file 4 Table S4.** Ionizing radiation and cancer risks sensitivity analysis

**Additional file 5 Table S5.** Meta regression analysis

**Additional file 6 Fig. S1.** Bubble plot depicting the relationship between “dose” as radiation to CT scans and “response” as cancer risks

**Additional file 7 Fig. S2.** Forest plot of cancer risk at different doses from CT scans

**Additional file 8 Fig. S3.** Forest plot of cancer risks at radiation doses from CT radiation exposure in age groups (A: <45 years; B: 45 to 65 years)

**Additional file 9 Video.** The association of cumulative ionizing radiation from CT scans with pertinent cancer risks in adults. (.mp4) (The source of the images included in the video are original.)

## Abbreviations

LAR: Lifetime attribution risk
OR: Odds ratio
CI: Confidence interval
BEIR: Biological Effects of Ionizing Radiation
Lbr: Lifetime baseline risk
RR: Relative risk
HBV: Hepatitis B virus
HPV: Human papilloma virus
HCV: Hepatitis C virus
LNT: Linear non-threshold
HDI: Human development index
