# Supplementary material for: CT Scans and Cancer Risks: A Systematic Review and Dose-response Meta-analysis
Source: BMC Cancer. 2022 Nov 30;22:1238. doi: 10.1186/s12885-022-10310-2 (PMC9710150; doi:10.1186/s12885-022-10310-2)
Supplement: Supplementary file 1 — Additional file 1 Table S1. Search strategy and results [file 12885_2022_10310_MOESM1_ESM.docx]

**Table S1.** Search strategy and results

| Database | Step | Search algorithm | Items founded |
| --- | --- | --- | --- |
| PubMed | #1 | tumour[All Fields] | 4220922 |
|  | #2 | neoplasms[All Fields] | 3512357 |
|  | #3 | tumor[All Fields] | 4220922 |
|  | #4 | cancer[All Fields] | 4314429 |
|  | #5 | #1 OR #2 OR #3 OR #4 | 4858446 |
|  | #6 | adult[MeSH Terms] | 7408761 |
|  | #7 | major[All Fields] | 1931259 |
|  | #8 | #6 OR #7 | 8830070 |
|  | #9 | computed tomography[All Fields] | 581839 |
|  | #10 | computerized tomography[All Fields] | 470378 |
|  | #11 | computerized tomography, x ray[MeSH Terms] | 447211 |
|  | #12 | #9 OR #10 OR #11 | 592289 |
|  | #13 | radionuclide imaging[MeSH Terms] | 217895 |
|  | #14 | radiation[All Fields] | 955334 |
|  | #15 | emissions[All Fields] | 270437 |
|  | #16 | radioactive fallout[MeSH Terms] | 3863 |
|  | #17 | fallout[All Fields] | 6213 |
|  | #18 | #13 OR #14 OR #15 OR #16 OR #17 | 1337759 |
|  | #19 | #5 AND #8 AND #12 AND #18 | 35952 |
| Embase | #1 | (tumour or neoplasms or tumor or cancer).mp. | 5151370 |
|  | #2 | (adult or major).mp. | 11353610 |
|  | #3 | (computerized tomography, x ray.mp. or Tomography, X-Ray Computed or CT or computerized tomography or computed tomography).mp. | 1519450 |
|  | #4 | (Radiation Injuries/ or Radiation/ or Radiation Exposure/ or Radiation Effects/ or Radiation, Ionizing/).mp. | 210609 |
|  | #5 | #1 and #2 and #3 and #4 | 3802 |
| Cochrane Library | #1 | MeSH descriptor: [adult] explode all trees | 459648 |
|  | #2 | MeSH descriptor: [tomography, X-Ray Computed] explode all trees | 5000 |
|  | #3 | MeSH descriptor:[Radionuclide Lmaging] explode all trees | 4603 |
|  | #4 | MeSH descriptor:[Radioactive Fallout] explode all trees | 4 |
|  | #5 | (tumour) OR (neoplasms) OR (tumor) OR (cancer) | 210657 |
|  | #6 | (major) | 296352 |
|  | #7 | (computed tomography) OR (computerized tomography) | 17372 |
|  | #8 | (radiation) OR (emissions) RO (fallout) | 30871 |
|  | #9 | #2 OR #3 | 687896 |
|  | #10 | #4 OR #5 | 17495 |
|  | #11 | #6 OR #7 OR #8 | 35220 |
|  | #12 | #1 AND #9 AND #10 AND #11 | 994 |
| Medline | #1 | (tumour or neoplasms or tumor or cancer).mp. | 3620303 |
|  | #2 | (adult or major).mp. | 6702116 |
|  | #3 | (computerized tomography, x ray.mp. or Tomography, X-Ray Computed or CT or computerized tomography or computed tomography).mp. | 627304 |
|  | #4 | Radiation Injuries/ or Radiation/ or Radiation Exposure/ or Radiation Effects/ or Radiation, Ionizing/ | 82842 |
|  | #5 | #1 and #2 and #3 and #4 | 841 |
| Web of science | #1 | ALL=(tumour OR neoplasms OR tumor OR cancer) | 4377659 |
|  | #2 | TS= computerized tomography, x ray OR ALL= computed tomography OR ALL= computerized tomography | 283305 |
|  | #3 | TS= (radioactive fallout OR radionuclide imaging) | 11005 |
|  | #4 | ALL=( radiation OR emissions OR fallout) | 1870909 |
|  | #5 | TS=adult OR ALL=major | 3321113 |
|  | #6 | #3 OR #4 | 1876932 |
|  | #7 | #6 AND #5 AND #2 AND #1 | 2355 |
| Clinicaltrials.gov | #1 | Cancer | 46123 |
|  | #2 | Neoplasm | 49053 |
|  | #3 | Tumor | 12945 |
|  | #4 | Malignancy | 3008 |
|  | #5 | Oncology | 1240 |
|  | #6 | Neoplasia | 578 |
|  | #7 | Neoplastic syndrome | 325 |
|  | #8 | Neoplastic disease | 20 |
|  | #9 | #1 OR #2 OR #3 OR #4 OR #5 OR #6 OR #7 OR #8 | 37370 |
|  | #10 | Adult | 23472 |
|  | #11 | Radiation Exposure | 113 |
|  | #12 | Radiation Injuries | 355 |
|  | #13 | Emissions | 1362 |
|  | #14 | radionuclide imaging | 1338 |
|  | #15 | #11 OR #12 OR #13 OR #14 | 2992 |
|  | #16 | Computed Tomography | 1150 |
|  | #17 | CT Scans | 298 |
|  | #18 | computerized tomography | 10 |
|  | #19 | Computerized axial tomography | 1 |
|  | #20 | Tomodensitometry | 8 |
|  | #21 | CAT scan | 7 |
|  | #22 | #16 OR #17 OR #18 OR #19 OR #20 OR #21 | 1530 |
|  | #23 | #9 AND #10 AND #15 AND #22 | 14 |
| Springer Link | #1 | ‘tumour OR neoplasms OR tumor OR cancer’ | 1583937 |
|  | #2 | ‘computerized tomography, x ray OR computed tomography OR computerized tomography’ | 22707 |
|  | #3 | 'radioactive fallout OR radionuclide imaging OR radiation exposure' | 11987 |
|  | #4 | ‘radiation OR emissions OR fallout’ | 1712019 |
|  | #5 | ‘adult OR major’ | 4602489 |
|  | #6 | #1 AND #2 AND #3 AND #4 AND #5 | 699 |
