# Supplementary material for: CT Scans and Cancer Risks: A Systematic Review and Dose-response Meta-analysis
Source: BMC Cancer. 2022 Nov 30;22:1238. doi: 10.1186/s12885-022-10310-2 (PMC9710150; doi:10.1186/s12885-022-10310-2)
Supplement: Supplementary file 2 — Additional file 2 Table S2. Contacting authors for additional information [file 12885_2022_10310_MOESM2_ESM.docx]

**Table S2.** Contacting authors for additional information

| Paper | Data requested | Response | Data provided |
| --- | --- | --- | --- |
| Meulepas,^1^ et al. 2018 | Primary data on the incidence of colorectal, lung, and breast cancer among 18- to 89-year-olds who received at least one CT scan between 1994 and 2014. | No response^a^ | None |
| Cohen,^2^ et al 2017 | Participants and cancer data were grouped by ≤15 mSv, 15-35 mSv, 35-55 mSv, and ≥55 mSv. | No response^a^ | None |
| Wu, ^3^ et al 2014 | Raw data about the LAR chart data. | No response^a^ | None |
| Basea, ^4^ et al 2018 | Data on the cancer cases and participants aged 18-20 years. | No response^a^ | None |

^a^Non-responding authors contacted up to two times, one month apart.

**References:**

1. Meulepas JM, Hauptmann M, Lubin JH, Shuryak I, Brenner DJ. Is there Unmeasured Indication Bias in Radiation-Related Cancer Risk Estimates from Studies of Computed Tomography? *Radiat Res.* 2018;189(2):128-135. doi: 10.1667/RR14807.1

2. Cohen S, Liu A, Gurvitz M, et al. Exposure to Low-Dose Ionizing Radiation From Cardiac Procedures and Malignancy Risk in Adults With Congenital Heart Disease. *Circulation.* 2018;137(13):1334-1345. doi: 10.1161/CIRCULATIONAHA.117.029138

3. Wu TH, Lin WC, Chen WK, Chang YC, Hwang JJ. Predicting cancer risks from dental computed tomography. *J Dent Res.* 2015;94(1):27-35. doi: 10.1177/0022034514554226

4. Bosch de Basea M, Morina D, Figuerola J, et al. Subtle excess in lifetime cancer risk related to CT scanning in Spanish young people. *Environ Int.* 2018;120:1-10. doi: 10.1016/j.envint.2018.07.020
