# Supplementary material for: CT Scans and Cancer Risks: A Systematic Review and Dose-response Meta-analysis
Source: BMC Cancer. 2022 Nov 30;22:1238. doi: 10.1186/s12885-022-10310-2 (PMC9710150; doi:10.1186/s12885-022-10310-2)
Supplement: Supplementary file 3 — Additional file 3 Table S3A. Newcastle-Ottawa Quality Assessment Scale – Case–control studies. (.docx). Table S3B. Newcastle-Ottawa Quality Assessment Scale – Cohort studies [file 12885_2022_10310_MOESM3_ESM.docx]

# Table S3A. Newcastle-Ottawa Quality Assessment Scale – Case–control studies

| Studies | Is the case definition adequate? | Selection | Selection of Controls | Definition of Controls | Comparability | Ascertainment of exposure | Outcome | Non-response rate | Score(☆) |
| --- | --- | --- | --- | --- | --- | --- | --- | --- | --- |
|  |  | Representativeness of the case |  |  | Comparability of cases and controls on the basis of the design or analysis (study adjusts for age☆, sex☆) |  | Same method of ascertainment for cases and controls |  |  |
| Shao, ^8^ et al 2020 | ☆ | ☆ | ☆ | ☆ | ☆☆ | ☆ | ☆ |  | 8 |
| Davis, ^11^ 2011 | ☆ | ☆ | ☆ | ☆ | ☆ | ☆ | ☆ |  | 7 |
| *One score of the Newcastle-Ottawa Quality Assessment Scale. | | | | | | | | | |

# Table S3B. Newcastle-Ottawa Quality Assessment Scale – Cohort studies.

| Studies | Representativeness of the exposed cohort | Selection | Ascertainment of exposure | Demonstration that outcome of interest was not present at start of study | Comparability | Assessment of outcome | Outcome | Adequacy of follow up of cohorts | Score(☆) |
| --- | --- | --- | --- | --- | --- | --- | --- | --- | --- |
|  |  | Selection of the non-exposed cohort |  |  | Comparability of cohorts on the basis of the design or analysis (study adjusts for age☆, sex☆) |  | Was follow-up long enough for outcomes to occur |  |  |
| Burton, ^35^ et al 2018 | ☆ | ☆ | ☆ | ☆ | ☆ | ☆ | ☆ | ☆ | 8 |
| Nordenskjöld, ^10^ et al 2017 | ☆ | ☆ | ☆ | ☆ | ☆☆ | ☆ | ☆ | ☆ | 9 |
| Hung, ^36^ et al 2013 | ☆ | ☆ | ☆ | ☆ | ☆ | ☆ | ☆ | ☆ | 9 |
| Rampinelli, ^9^ et al. 2017 | ☆ |  | ☆ | ☆ |  | ☆ | ☆ | ☆ | 6 |
| Olsen, ^10^ et al 2014^53^ | ☆ |  | ☆ | ☆ |  | ☆ | ☆ | ☆ | 6 |
| Kritsaneepaiboon, ^37^ et al 2016 | ☆ |  | ☆ | ☆ |  | ☆ |  | ☆ | 5 |
| Griffey, ^38^ et al 2009 | ☆ |  | ☆ | ☆ |  | ☆ |  | ☆ | 5 |
| Einstein, ^39^ et al 2008 | ☆ |  | ☆ | ☆ |  |  |  | ☆ | 4 |
| Faletra, ^40^ et al 2010 | ☆ |  | ☆ | ☆ |  |  |  | ☆ | 4 |
| Niemann, ^41^ et al 2013 | ☆ |  | ☆ | ☆ |  |  |  | ☆ | 4 |
| Huang, ^42^ et al 2009 |  |  | ☆ | ☆ |  |  |  |  | 2 |
| Perisinakis, ^43^ et al 2015 |  |  | ☆ | ☆ |  |  |  | ☆ | 3 |
| Smith-Bindman, ^2^ et al 2009 | ☆ |  | ☆ | ☆ |  |  |  | ☆ | 4 |
| Sodickson, ^44^ et al 2009 | ☆ |  | ☆ | ☆ |  |  |  | ☆ | 4 |
| Huang, ^45^ et al 2009 |  |  | ☆ | ☆ |  |  |  |  | 2 |
| Huda, ^46^ et al 2010 | ☆ |  | ☆ | ☆ |  |  |  |  | 3 |
| Perisinakis, ^47^ et al 2012 |  |  | ☆ | ☆ |  |  |  | ☆ | 3 |
| Einstein, ^48^ et al 2007 |  |  | ☆ | ☆ |  |  |  |  | 2 |
| Kim, ^49^ et al 2009 | ☆ |  | ☆ | ☆ |  |  |  | ☆ | 4 |
| Majer, ^50^ et al 2018 |  |  | ☆ | ☆ |  |  |  |  | 2 |
| Salibi, ^51^ et al 2014 | ☆ |  | ☆ | ☆ |  |  | ☆ | ☆ | 5 |
| Shah, ^52^ et al 2013 | ☆ |  | ☆ | ☆ |  |  | ☆ | ☆ | 5 |
| Wylie,^53^ et al 2018 | ☆ |  | ☆ | ☆ |  |  |  |  | 3 |
| ☆One score of the Newcastle-Ottawa Quality Assessment Scale | | | | | | | | | |
