# Supplementary material for: CT Scans and Cancer Risks: A Systematic Review and Dose-response Meta-analysis
Source: BMC Cancer. 2022 Nov 30;22:1238. doi: 10.1186/s12885-022-10310-2 (PMC9710150; doi:10.1186/s12885-022-10310-2)
Supplement: Supplementary file 4 — Additional file 4 Table S4. Ionizing radiation and cancer risks sensitivity analysis [file 12885_2022_10310_MOESM4_ESM.docx]

# Table S4. Ionizing radiation and cancer risks sensitivity analysis^a^

| Study | Ι*^2^* % | Linear analyses  OR (95% CI) |
| --- | --- | --- |
|  |  |  |
| Burton, ^35^ et al 2018 | 99.59 | 6.59 (3.85 to 11.27) |
| Nordenskjöld, ^10^ et al 2017 | 99.63 | 6.30 (3.59 to 11.05) |
| Hung, ^36^ et al 2013 | 99.62 | 6.43 (3.70 to 11.17) |
| Olsen, ^54^ et al 2014 | 99.62 | 6.39 (3.67 to 11.14) |
| Shao, ^8^ et al 2020 | 99.61 | 6.35 (3.63 to 11.11) |
| Davis, ^11^ et al 2011 | 99.62 | 6.36 (3.64 to 11.11) |
| Rampinelli, ^9^ et al 2017 | 99.63 | 6.04 (3.41 to 10.70) |
| Kritsaneepaiboon, ^37^ et al 2016 | 99.61 | 5.98 (3.46 to 10.35) |
| Griffey, ^38^ et al 2009 | 99.58 | 5.37 (3.17 to 9.09) |
| Einstein, ^39^ et al 2008 | 99.64 | 6.03 (3.41 to 10.68) |
| Faletra, ^40^ et al 2010 | 99.59 | 5.67 (3.24 to 9.95) |
| Niemann, ^41^ et al 2013 | 99.62 | 6.10 (3.45 to 10.79) |
| Huang, ^42^ et al 2009 | 99.53 | 5.48 (3.18 to 9.43) |
| Perisinakis, ^43^ et al 2015 | 99.63 | 6.19 (3.51 to 10.91) |
| Smith-Bindman, ^2^ et al 2009 | 99.64 | 5.90 (3.34 to 10.45) |
| Sodickson, ^44^ et al 2009 | 99.64 | 5.85 (3.31 to 10.34) |
| Huang, ^45^ et al 2009 | 99.58 | 5.51 (3.19 to 9.51) |
| Huda, ^46^ et al 2010 | 99.63 | 5.62 (3.22 to 9.80) |
| Perisinakis, ^47^ et al 2012 | 99.64 | 5.92 (3.34 to 10.47) |
| Einstein, ^48^ et al 2007 | 99.62 | 5.80 (3.28 to 10.23) |
| Kim, ^49^ et al 2009 | 99.60 | 6.48 (3.74 to 11.21) |
| Majer, ^50^ et al 2018 | 99.62 | 5.63 (3.22 to 9.84) |
| Salibi, ^51^ et al 2014 | 99.62 | 5.68 (3.24 to 9.96) |
| Shah, ^52^ et al 2013 | 99.60 | 5.55 (3.20to 9.64) |
| Wylie,^53^ et al 2018 | 99.61 | 6.06 (3.42 to 10.73) |

Abbreviations: OR, odds ratio; CI, confidence interval.

^a^ Sensitivity analyses were performed by excluding one study at a time from the meta-analysis to identify the effect of any one individual study.
