# Supplementary material for: CT Scans and Cancer Risks: A Systematic Review and Dose-response Meta-analysis
Source: BMC Cancer. 2022 Nov 30;22:1238. doi: 10.1186/s12885-022-10310-2 (PMC9710150; doi:10.1186/s12885-022-10310-2)
Supplement: Supplementary file 5 — Additional file 5 Table S5. Meta regression analysis [file 12885_2022_10310_MOESM5_ESM.docx]

# Table S5. Meta regression analysis

**Table S5a.** Radiation dose

| _meta_es | Coef. | Std. err. | z | P | 95% CI |
| --- | --- | --- | --- | --- | --- |
| dose | 1.2980 | 0.2361 | 5.50 | 0.000 | 0.8352 to 1.7607 |
| cons | -0.5920 | 0.5074 | -1.17 | 0.243 | -1.5865 to 0.4023 |

Z, value for the z statistic; Coef, coefficient; Std. Err, Standard error; P, p-value; CI, Confidence Interval.

**Table S5b.** CT sites

| _meta_es | Coef. | Std. err. | z | P | 95% CI |
| --- | --- | --- | --- | --- | --- |
| sites | 1.4279 | 0.5032 | 2.84 | 0.005 | 0.4417 to 2.4141 |
| cons | -0.2106 | 0.7468 | -0.28 | 0.778 | -1.6742 to 1.2530 |

Z, value for the z statistic; Coef, coefficient; Std. Err, Standard error; P, p-value; CI, Confidence Interval.

**Table S5c.** Actual / Estimate

| _meta_es | Coef. | Std. err. | z | P | 95% CI |
| --- | --- | --- | --- | --- | --- |
| data | 2.1421 | 0.4989 | 4.29 | 0.000 | 1.1642 to 3.1201 |
| cons | -1.9812 | 0.9039 | -2.19 | 0.028 | -3.7523 to -0.2095 |

Z, value for the z statistic; Coef, coefficient; Std. Err, Standard error; P, p-value; CI, Confidence Interval.

**Table S5d.** Leukaemia / Non Leukaemia

| _meta_es | Coef. | Std. err. | z | P | 95% CI |
| --- | --- | --- | --- | --- | --- |
| leukaemia | -1.1184 | 0.8073 | -1.39 | 0.166 | -2.7007 to 0.4639 |
| cons | 2.8380 | 1.0599 | 2.68 | 0.007 | 0.7606 to 4.9153 |

Z, value for the z statistic; Coef, coefficient; Std. Err, Standard error; P, p-value; CI, Confidence Interval.

**Table S5e.** America / Non America

| _meta_es | Coef. | Std. err. | z | P | 95% CI |
| --- | --- | --- | --- | --- | --- |
| america | 0.7192 | 0.5557 | 1.29 | 0.196 | -0.3700 to 1.8084 |
| cons | 0.8187 | 0.8578 | 0.95 | 0.340 | -0.8625 to 2.5000 |

Z, value for the z statistic; Coef, coefficient; Std. Err, Standard error; P, p-value; CI, Confidence Interval.

**Table S5f.** Gender

| _meta_es | Coef. | Std. err. | z | P | 95% CI |
| --- | --- | --- | --- | --- | --- |
| gender | 0.1852 | 0.5043 | 0.37 | 0.713 | -0.8039 to 1.1736 |
| cons | 1.1402 | 0.7981 | 1.76 | 0.079 | -0.1627 to 2.9657 |

Z, value for the z statistic; Coef, coefficient; Std. Err, Standard error; P, p-value; CI, Confidence Interval.

**Table S5g.** Age

| _meta_es | Coef. | Std. err. | z | P | 95% CI |
| --- | --- | --- | --- | --- | --- |
| age | -0.0564 | 0.3772 | 0.15 | 0.881 | -0.6829 to 0.7956 |
| cons | 1.6355 | 0.7733 | 2.11 | 0.034 | 0.1198 to 3.1512 |

Z, value for the z statistic; Coef, coefficient; Std. Err, Standard error; P, p-value; CI, Confidence Interval.

**Table S5h.** Data before / after 2007

| _meta_es | Coef. | Std. err. | z | P | 95% CI |
| --- | --- | --- | --- | --- | --- |
| time | -0.1093 | 0.9022 | -0.12 | 0.904 | -1.8777 to 1.6589 |
| cons | 1.4506 | 1.3263 | 1.09 | 0.274 | -1.1491 to 4.0502 |

Z, value for the z statistic; Coef, coefficient; Std. Err, Standard error; P, p-value; CI, Confidence Interval.

**Table S5j.** Quality scores

| _meta_es | Coef. | Std. err. | z | P | 95% CI |
| --- | --- | --- | --- | --- | --- |
| quality | -0.6660 | 0.5583 | -1.19 | 0.233 | -1.7604 to 0.4283 |
| cons | 2.7472 | 0.8497 | 3.23 | 0.001 | 1.0819 to 4.4127 |

Z, value for the z statistic; Coef, coefficient; Std. Err, Standard error; P, p-value; CI, Confidence Interval.
