# Supplementary figures and images for: CT Scans and Cancer Risks: A Systematic Review and Dose-response Meta-analysis
Source: BMC Cancer. 2022 Nov 30;22:1238. doi: 10.1186/s12885-022-10310-2 (PMC9710150; doi:10.1186/s12885-022-10310-2)

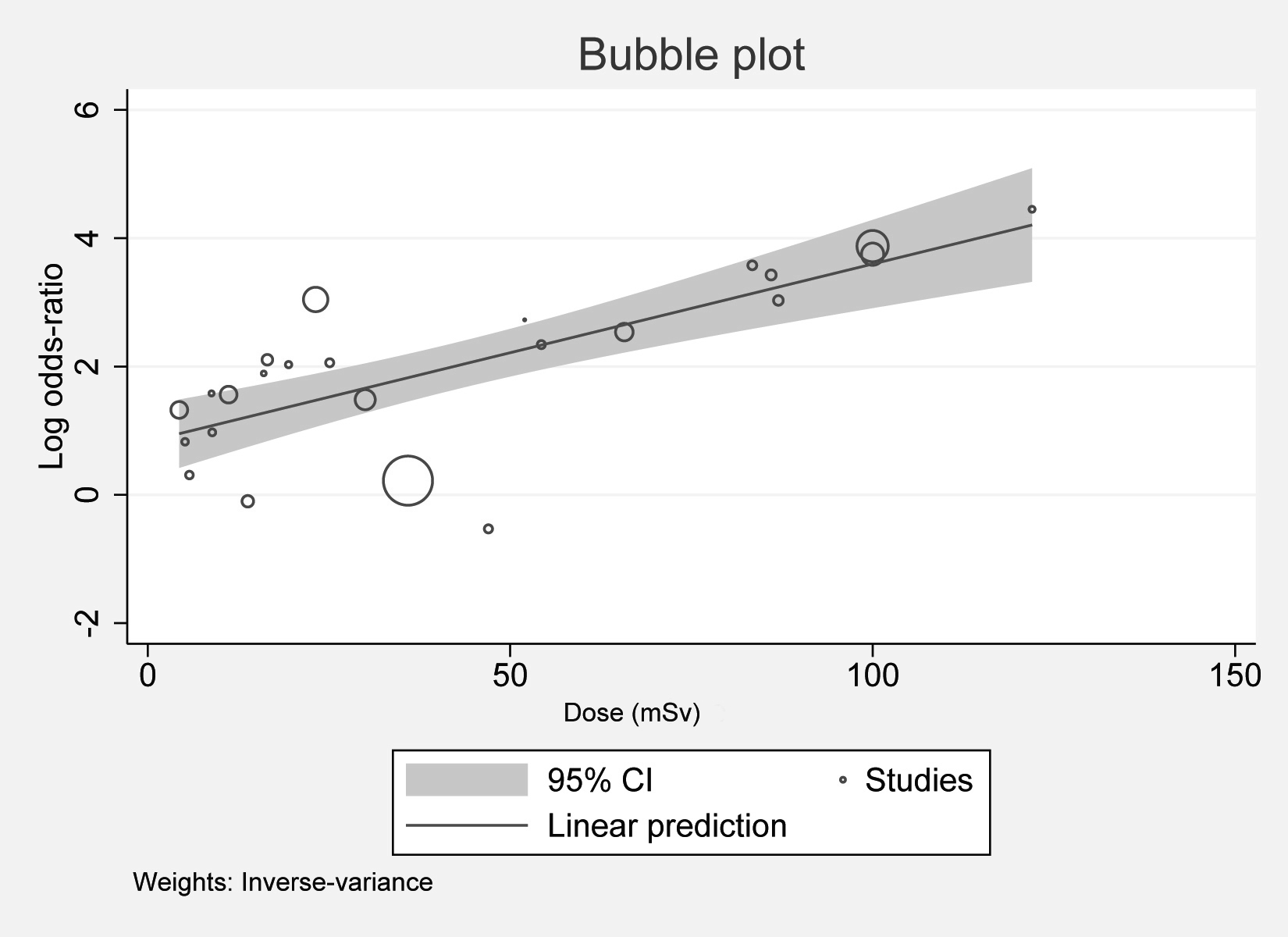

Supplement: Supplementary file 6 — Additional file 6 Fig. S1. Bubble plot depicting the relationship between “dose” as radiation to CT scans and “response” as cancer risks [file 12885_2022_10310_MOESM6_ESM.jpg]

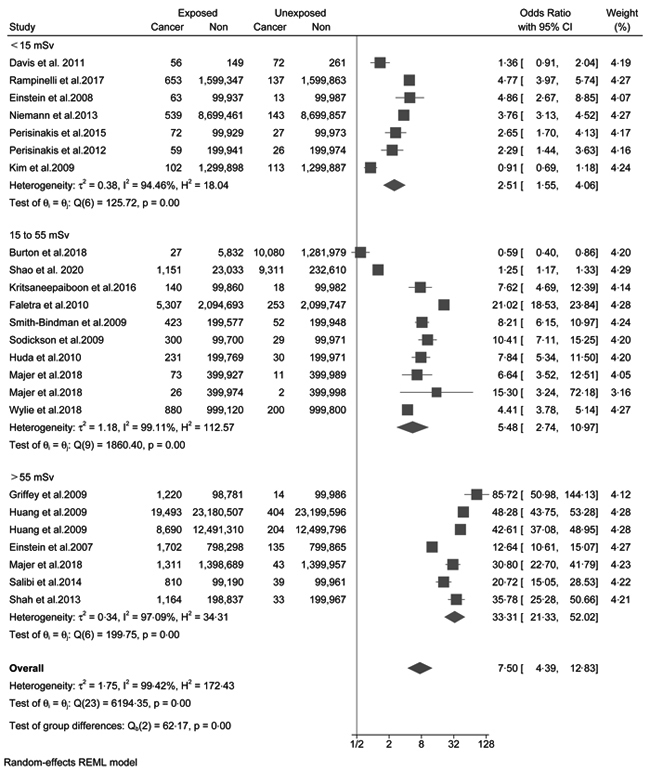

Supplement: Supplementary file 7 — Additional file 7 Fig. S2. Forest plot of cancer risk at different doses from CT scans [file 12885_2022_10310_MOESM7_ESM.jpg]

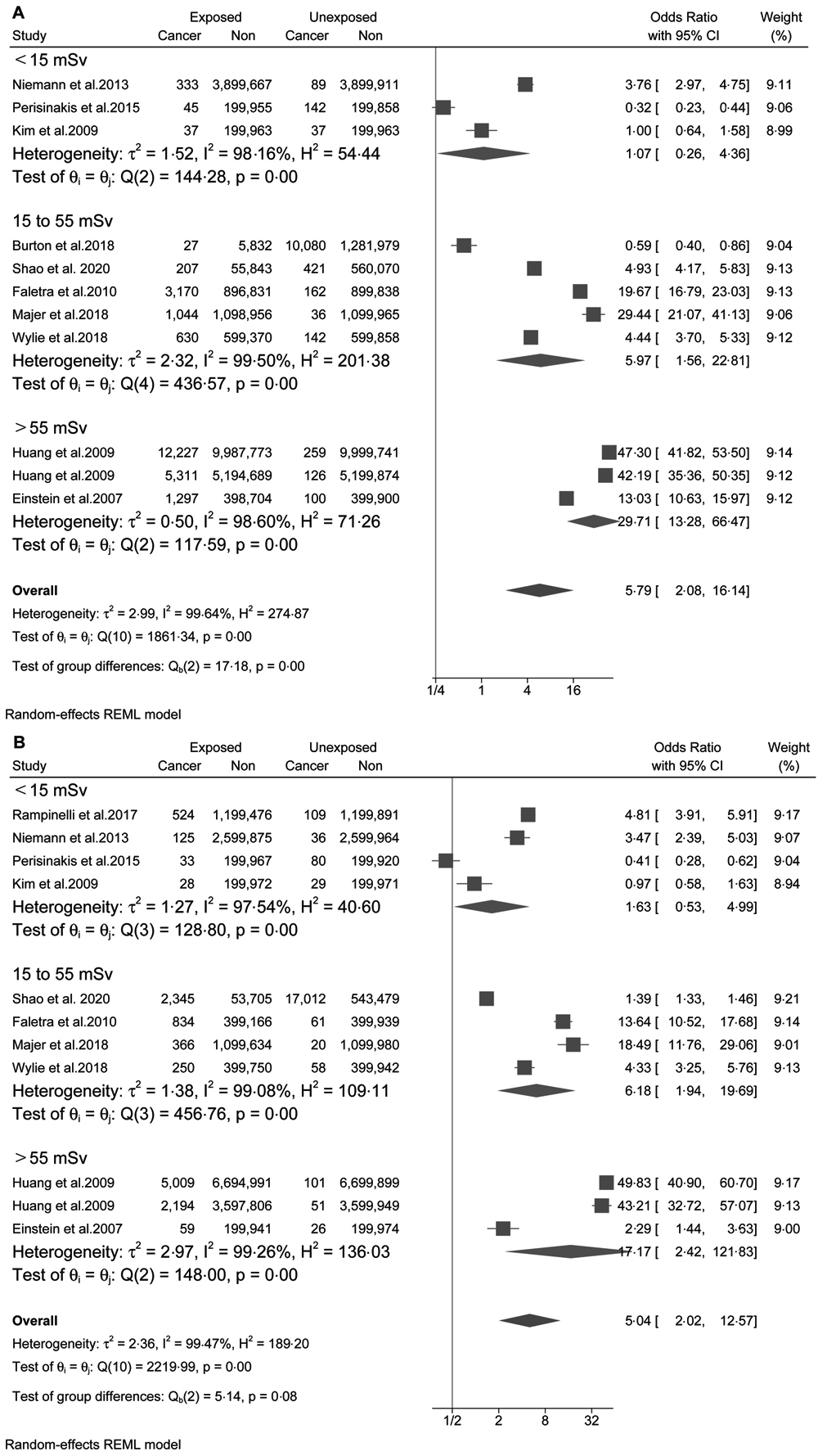

Supplement: Supplementary file 8 — Additional file 8 Fig. S3. Forest plot of cancer risks at radiation doses from CT radiation exposure in age groups (A: <45 years; B: 45 to 65 years) [file 12885_2022_10310_MOESM8_ESM.jpg]
